# Toward A Pre-disease State-centered New Paradigm in Multi-omics Research

**DOI:** 10.1093/gpbjnl/qzag016

**Published:** 2026-03-01

**Authors:** Cheng Lu (陆铖), Yan Li (李艳), Quansheng Du (杜全生)

**Affiliations:** Department of Interdisciplinary Sciences, National Natural Science Foundation of China, Beijing 100085, China; Department of Radiology, Guangdong Provincial People’s Hospital, Guangdong Academy of Medical Sciences, Southern Medical University, Guangzhou 510080, China; Department of Interdisciplinary Sciences, National Natural Science Foundation of China, Beijing 100085, China; National Local Joint Engineering Research Center for Precision Surgery & Regenerative Medicine, The First Affiliated Hospital of Xi’an Jiaotong University, Xi’an 710061, China; Department of Interdisciplinary Sciences, National Natural Science Foundation of China, Beijing 100085, China

Multi-omics technologies offer robust approaches for investigating genotype–phenotype relationships, with potential to elucidate the biological mechanisms across micro-to-macro scales [1,2]. Nevertheless, the intrinsic complexity and dynamic interconnectivity of biological systems reveal core challenges, including the integration and causal inference of biological information [3,4]. Presently, critical data remain insufficient, such as the dearth of ultrastructural data that delineate the spatiotemporal dynamics of cellular components [5]. Furthermore, a standardized and artificial intelligence (AI)-ready data ecosystem has not yet been established [6]. There is a paucity of large-scale models capable of processing and integrating multi-dimensional biological information [7]. In this perspective, we propose a conceptual framework centered on the “pre-disease state” in health and disease research. In this context, we define the pre-disease state as a critical transition phase within nonlinear biological systems — characterized by systemic instability and coordinated molecular network fluctuations that occur at the tipping point prior to irreversible disease commitment. This perspective articulates a synthesis of two complementary dimensions: by bridging theoretical systems biology with practical multi-omics strategy, we aim to not only characterize the system-level criticality, dynamic transitions, and networked reorganization during early disease progression, but also to outline strategic directions for the next generation of multi-omics methodologies capable of capturing these shifts.

## Development and representative findings in multi-omics research

### Data source

We first conducted a bibliometric analysis using literature retrieved from the Web of Science (WoS) Core Collection database (Clarivate Analytics). With the rapid development of high-throughput technologies, AI, and multimodal data acquisition platforms, the scope of omics research has expanded beyond traditional molecular layers to incorporate functional, structural, environmental, and clinical data. To ensure comprehensive and relevant retrieval, the search strategy centered on the term root “omics”, while excluding commonly confounding terms using the Boolean query:


TS=(*omics) NOT  TS=(*economics  OR  *comics  OR  *atomics  OR  *telecomics  OR  *nanomics)

The search was limited to peer-reviewed original research articles and review papers published between 2015 and 2024. The literature search was conducted in the WoS Core Collection on November 4, 2025. We obtained 341,095 publications prior to excluding items unrelated to biomedical sciences, including those from political science, economics, or management. After applying the full set of inclusion and exclusion criteria, a total of 261,288 publications remained for analysis. Bibliographic records were exported and analyzed using VOSviewer [8] for visualization-based bibliometric mapping. The data collection and analysis processes are shown in **Figure 1**A, and the list of cited references included in the co-citation network is shown in Table S1. The resulting analytical outputs are summarized in the following sections.

**Figure 1:**
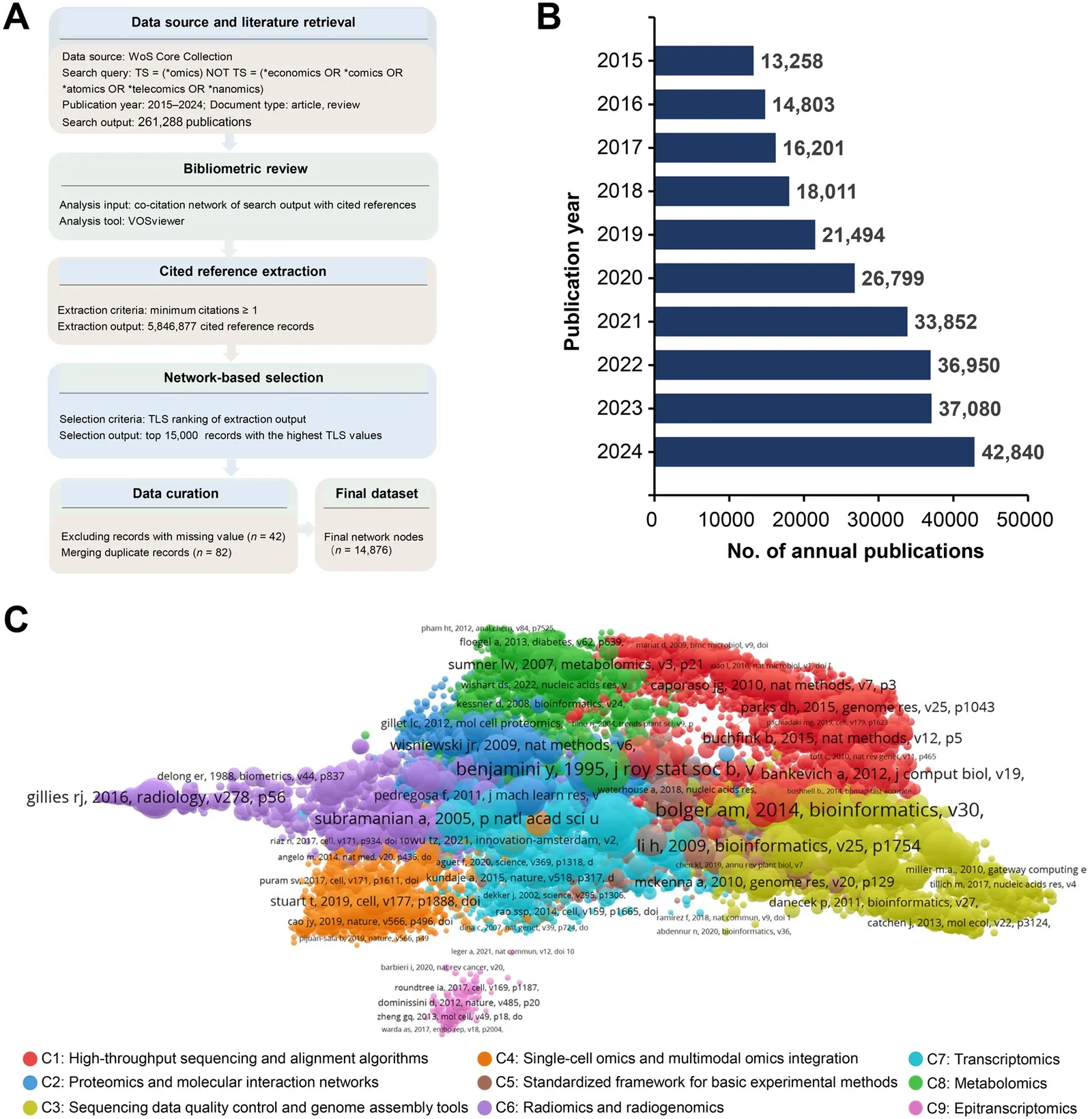
Trends and themes of the multi-omics research landscape **A**. Workflow of literature retrieval and co-citation network construction. Source documents were retrieved from the WoS Core Collection using a predefined search strategy. Reference lists of the 261,288 source documents were aggregated, yielding 5,846,877 cited reference records, including duplicates. All cited reference records met the minimum citation threshold (≥ 1) and were included in the initial co-citation analysis. TLS, defined as the sum of the links of a node with all other nodes, was calculated for each cited reference. The top 15,000 references were selected as network nodes to ensure visualization clarity and computational feasibility. After duplicate merging and removal of records with missing information, 14,876 unique cited references remained, forming the final co-citation network. **B**. Annual publication volume for omics-related research from 2015 to 2024 based on records retrieved from the WoS Core Collection. **C**. Visualized co-citation network generated based on the 14,876 unique cited reference nodes. Each node represents a publication, with node size proportional to the number of citations received. Node colors indicate different research themes (clusters). Labels are automatically positioned to enhance readability and minimize overlap. A complete list of cited references included in the network is provided in Table S1. WoS, Web of Science; TLS, total link strength.

### Analysis of development trends in omics research

#### Publication volume trends

As shown in Figure 1B, the number of omics-related publications has increased markedly over the past decade, rising from 13,258 publications in 2015 to an estimated 42,840 publications in 2024, representing a 223% increase. This corresponds to an average annual growth rate of approximately 13.6%. From 2015 to 2019, publication counts increased steadily, reflecting the growing adoption of high-throughput technologies and integrative analysis frameworks across biomedical research. A pronounced acceleration occurred between 2019 and 2021, coinciding with global scientific efforts. During this period, research activity expanded rapidly in areas such as viromics, immunomics, metabolomics, and host–pathogen multi-omics profiling. Growth stabilized between 2022 and 2023, reflecting a return to pre-pandemic research dynamics. The notable rise in 2024 suggests continued expansion and evolving research interests in multi-omics methodologies and applications.

#### Major advances and representative achievements

Co-citation analysis identified nine major thematic clusters that tracked the field’s evolution from foundational tools to phenotype-driven profiling (Figure 1C). Historically, early efforts focused on genomic alignment and statistical infrastructure, exemplified by tools like Basic Local Alignment Search Tool (BLAST) [9], Burrows-Wheeler Aligner (BWA) [10], and Bowtie [11], false discovery rate correction methods [12], and the completion of the Human Genome Project [13]. Technological optimization continued with high-speed protein alignment algorithms like DIAMOND (fast protein alignment tool) [14], which further accelerated analytical speeds. These foundational technologies rapidly expanded into specialized domains: epitranscriptomics revealed post-transcriptional regulatory mechanisms [15–19], while advances in mass spectrometry established robust workflows for proteomics and metabolomics [20,21]. This era also saw the development of essential resources, such as Cytoscape [22], the Human Protein Atlas [23], and databases like MetaboAnalyst [24] and Human Metabolome Database [25], alongside the formalization of microbiome modeling [26] and radiomics [27–29].

As multi-dimensional datasets accumulated, the research focus shifted toward integration and predictive intelligence. Advanced computational frameworks, including graph neural networks (GNNs) and deep learning, have enabled the transition from descriptive pattern recognition to causal inference [30]. Recent breakthroughs in single-cell and spatial multi-omics have been pivotal in resolving tissue heterogeneity [31], yielding critical insights into cancer subtypes [32], targeted therapy outcomes [33], immunotherapy responses [34], and complex disease mechanisms [35]. Most recently, cutting-edge tools have integrated GNNs with spatial transcriptomics for precise domain inference [36] and utilized generative large language models (*e.g.*, scGPT) to simulate cell state progression [37], marking a clear trajectory toward clinically aligned, mechanistic systems.

## Development trends and challenges

Life and health sciences are experiencing a major conceptual shift — from static, qualitative description toward dynamic, quantitative, and mechanistic understanding of biological systems. The field is transitioning from pattern recognition based on post hoc observations to real-time *in situ* characterization of biological evolution, placing new demands on current multi-omics research frameworks and analytical strategies.

A primary challenge is the lack of mathematical and theoretical models capable of capturing dynamic biological evolution. Although biological systems are inherently dynamic, most multi-omics studies still rely on single-time-point, cross-sectional comparisons. These “static snapshots” limit our ability to model temporal trajectories or quantify continuous system fluctuations. For example, during the prediabetic stage, blood glucose levels may remain within the clinically normal range, while nonlinear perturbations in insulin sensitivity, lipid metabolism, and regulatory feedback networks have already begun. Existing analytical methods are not yet capable of accurately modeling the rate, direction, or stochasticity of these micro-scale physiological transitions [38].

A second challenge lies in quantifying biological states from high-dimensional, nonlinear, multimodal datasets. Multi-omics data vary widely in scale, measurement format, biological meaning, and intrinsic technical noise. Because biological systems exist in dynamic equilibrium across varying spatial and temporal contexts, current computational approaches remain insufficient for integrating heterogeneous datasets to derive stable and biologically meaningful state metrics. For example, multi-layer omics data — including transcriptomics, proteomics, metabolomics, and microbiomics — exhibit nonlinear age-dependent shifts [39], indicating that biological states are neither linear nor stationary but instead undergo discontinuous transitions. Yet most current analytical frameworks still rely on assumptions of linearity, Gaussian distributions, or fixed-state models, limiting their suitability for dynamic biological inference. Although deep learning approaches show promise for modeling these nonlinear, multimodal datasets, such methods remain in an early developmental stage and lack robust theoretical grounding and interpretability [40].

A third major challenge is to distinguish the causal mechanisms driving disease transitions from statistical correlations. While multi-omics datasets are powerful tools for identifying associated molecular features, the presence of confounding factors complicates causal inference. For example, an analysis of host genomic variation and gut microbiome composition in 7738 individuals from the Dutch population revealed widespread correlation between host genotype and microbial taxa. However, disentangling true genetic effects from environmental confounders — such as diet, lifestyle, and geographic exposure — remains statistically challenging [41]. Without causal inference, the identification of actionable therapeutic targets, biomarkers, or intervention points remains uncertain.

Collectively, challenges stemming from data heterogeneity, sparsity, noise, and limited temporal resolution hinder our ability to characterize biological evolution, quantify transient states, and infer causal mechanisms. Addressing these limitations will require the development of new theoretical frameworks, computational strategies, and experimental designs that bridge the fundamental biological research with clinical application, particularly in the context of early disease detection and precision intervention [42].

## Exploring new paradigms in multi-omics research

To fundamentally overcome the limitations of static, correlation-driven approaches, it is necessary to move beyond the current “snapshot” paradigm. The core challenges identified above — specifically the difficulty in distinguishing causal disease signals from high-dimensional stochastic noise — stem from overlooking the intermediate dynamical regime that bridges health and disease. In this critical phase, system stability erodes, and molecular networks exhibit coordinated fluctuations that conventional static models frequently misinterpret as noise or discard as heterogeneity. Therefore, adopting a pre-disease state perspective is not merely an alternative research direction, but a necessary methodological prerequisite. It provides the essential theoretical context to decode these dynamic instabilities, thereby transforming multi-omics from a descriptive retrospective tool into a predictive framework capable of interceptive intervention. Therefore, in this perspective, we propose a research paradigm centered on the pre-disease state, emphasizing early biological transitions before clinical symptoms emerge. This approach focuses on the critical transition state, in which multilevel, cross-scale, and nonlinear interactions dynamically emerge within living systems. Its objectives include deciphering regulatory mechanisms across biological scales, characterizing multivariate synergies, developing theoretical frameworks for nonlinear biological systems, and establishing interdisciplinary analytical strategies and technologies capable of tracking dynamic biological processes. Collectively, this paradigm represents a potential breakthrough pathway toward overcoming existing methodological barriers in multi-omics research [43]. **Figure 2** illustrates the overview of the pre-disease state-centered new paradigm.

**Figure 2:**
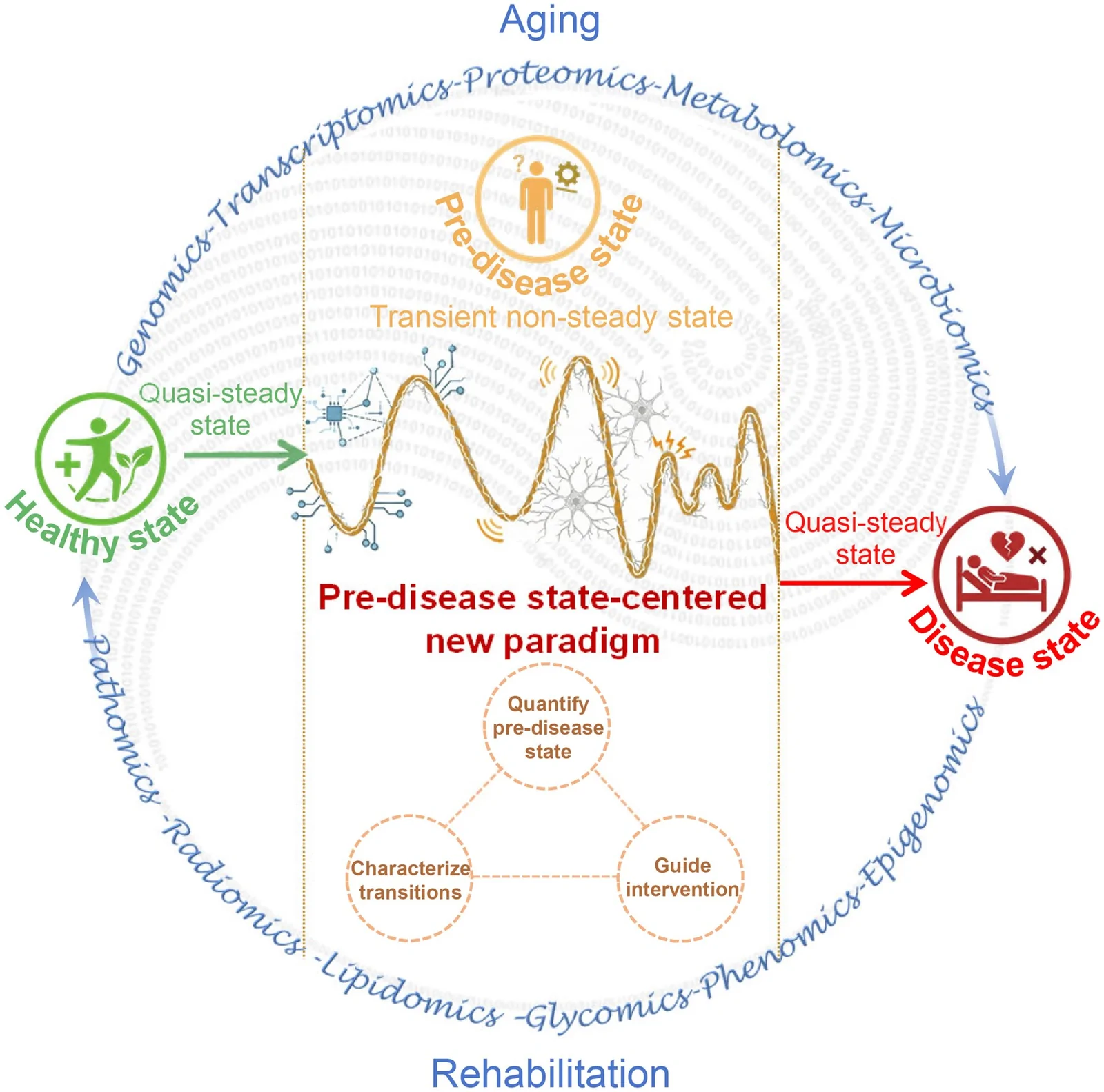
A pre-disease state-centered multi-omics new paradigm The pre-disease state represents an evolutionarily conserved transient non-steady state, which fluctuates between the quasi-steady healthy and disease states. This critical non-equilibrium phase is ubiquitous, occurring alongside dynamic biological trajectories such as aging and rehabilitation. The novel research strategy empowers multi-omics studies by enabling systematic mapping of biological complexity through a transdisciplinary framework. It rests on three pillars: quantification of the pre-disease state, characterization of critical transitions, and delivery of precision interventions.

The first core element of this paradigm involves the characterization of critical transition states within key biological processes. The pre-disease state can be naturally framed as a critical transition state in nonlinear biological systems, providing a theoretical foundation for early detection and system-level intervention. The pre-disease state is an empirically tractable regime increasingly validated by longitudinal multi-omics studies. For instance, longitudinal multi-omics profiling of human aging has revealed distinct nonlinear molecular shifts that precede phenotypic decline, effectively mapping a physiological “state transition” [39]. In metabolic research, deep phenotyping has identified specific sub-phenotypes of prediabetes defined by distinct multi-omics signatures, demonstrating that the pre-disease phase is a heterogeneous state with specific molecular trajectories rather than a silent latency [38]. Similarly, large-scale proteomic initiatives have identified biomarker cascades in neurodegenerative diseases that diverge from healthy baselines years before cognitive impairment [35], offering a concrete window for interception. Furthermore, advances in spatial transcriptomics have begun to characterize “primed” tissue microenvironments — such as specific fibroblast niches in immune-hot versus immune-cold tumors — that may facilitate eventual pathological progression [44]. These diverse empirical findings confirm the existence of measurable intermediate states across varying disease contexts. From the perspective of nonlinear dynamical systems, the pre-disease state represents a critical threshold between stability and instability [45]. Although phenotypic changes may be minimal at this stage, the underlying system stability is markedly weakened, rendering traditional controlled study designs insufficient for effective detection [46]. Therefore, a new framework is needed to dynamically quantify the transition state. From an informatics perspective, the pre-disease state is associated with increasing network complexity, which can be quantified using network flow entropy. To manage this high-dimensional complexity, AI-driven unsupervised learning — specifically graph autoencoder and deep embedding clustering [47] — can be employed to integrate and dimensionally reduce large-scale multi-omics data. Following this integration, dynamic Bayesian networks and differential equation-based models serve to quantify system resilience and recovery potential. Crucially, these computational models leverage time-series multi-omics data to infer interaction strengths and directional dynamics across molecular networks [48].

The second component involves the discovery of dynamic network biomarkers (DNBs) and the quantitative identification of pre-disease states. The defining characteristic of the pre-disease state is the emergence of coordinated dynamic fluctuations at the network level. DNBs provide a computational tool to detect these early destabilizing signals before phenotypic manifestation [49]. Two major challenges persist: (1) extracting temporally resolved DNBs from high-dimensional data and (2) accurately distinguishing biological signals from noise. These challenges may be addressed by constructing causal network models using time-series multi-omics data. Constraint-based algorithms [*e.g.*, Peter-Clark (PC) algorithm] and score-based approaches [*e.g.*, Greedy Equivalence Search (GES)] enable systematic identification of key nodes involved in early cascade effects during the pre-disease transition [50]. Structural causal models (SCMs) and causal generative adversarial networks (CGANs) further allow simulation of dynamic regulatory paths and generate testable mechanistic hypotheses for experimental validation [51]. Together, these tools enable a transition from qualitative description to quantitative causal interpretation, supporting early intervention strategies.

The third component centers on developing early warning and intervention frameworks that translate pre-disease detection into precision medicine applications. Early intervention strategies targeting critical transition states may accelerate the evolution of preventive medicine from conceptual aspiration to practical implementation. From a pre-disease state perspective, early warning and intervention frameworks shift multi-omics research from retrospective description toward predictive and actionable system control. Methodologically, multimodal contrastive learning offers a robust strategy for alignment. This technique trains neural networks to map multi-scale omics data into a unified latent representational space, thereby facilitating cross-modal alignment and integrative interpretation [52]. Additionally, model interpretability frameworks — such as SHapley Additive exPlanations (SHAP) and Local Interpretable Model-agnostic Explanations (LIME) — combined with explainable AI facilitate the development of interpretable risk prediction models [53]. The ultimate objective is to construct a causally interpretable, hypothesis-driven multi-omics knowledge graph that links microscopic molecular perturbations to macroscopic phenotypic shifts, providing quantitative foundations for precision diagnosis, prevention, and intervention.

In conclusion, multi-omics research under the pre-disease state viewpoint offers the potential to construct a continuous, dynamic framework describing the transition from a healthy state to a pre-disease state or eventually to a clinical disease state. This paradigm introduces new strategies for defining critical transition thresholds, elucidating the underlying regulatory mechanisms, and enabling cross-scale causal inference. By integrating data-driven computational approaches with mechanistic biological understanding, research on the pre-disease state may overcome current bottlenecks and accelerate progress from theoretical exploration to clinical translation. Ultimately, this shift will support the evolution from a passive, treatment-centered medical model to an active, prevention-oriented health framework, opening new avenues for both foundational biological discovery and precision medicine.

## CRediT author statement

**Cheng Lu:** Writing – original draft, Investigation, Visualization. **Yan Li:** Writing – original draft, Data curation, Formal analysis. **Quansheng Du:** Conceptualization, Supervision, Project administration, Writing – review & editing. All authors have read and approved the final manuscript.

## Competing interests

The authors have declared no competing interests.

## Supplementary material

Supplementary material is available at *Genomics, Proteomics & Bioinformatics* online (https://doi.org/10.1093/gpbjnl/qzag016).

## Supplementary Material

qzag016_Supplementary_Data
