# Supplementary material for: Toward A Pre-disease State-centered New Paradigm in Multi-omics Research
Source: Genomics Proteomics Bioinformatics. 2026 Mar 1;24(3):qzag016. doi: 10.1093/gpbjnl/qzag016 (PMC13630448; doi:10.1093/gpbjnl/qzag016)
Supplement: Supplementary file 1 [file qzag016_supplementary_data.zip › Supplementary material captions.docx]

**Supplementary material**

**Table S1 List of cited references included in the co-citation network (n = 14,876)**
